# Supplementary material for: Design of a Forest Fire Early Alert System through a Deep 3D-CNN Structure and a WRF-CNN Bias Correction
Source: Sensors (Basel). 2022 Nov 14;22(22):8790. doi: 10.3390/s22228790 (PMC9693021; doi:10.3390/s22228790)
Supplement: Supplementary file 1 [file sensors-22-08790-s001.zip › sensors-2012928-supplementary.pdf]

Design of a Forest Fire Early Alert System through a Deep 3D-CNN Structure and a WRF-CNN Bias Correction

The following document contains supplementary material from the article "**Design of a Forest Fire Early Alert System through a Deep 3D-CNN Structure and a WRF-CNN Bias Correction**".

Section S1. 3D neural networks characteristics

Table S1. 3D-CNN structure and characteristics. Notice that this network forecasts the wind components and the 2m temperature

Type of Layer	Output	Characteristics
Convolutional 3D	(1, 7, 6, 32)	Kernel size = (56,2,2)
MaxPooling 3D	(1, 3, 3, 32)	-
Batch Normalization	(1, 3, 3, 32)	-
Convolutional 3D	(1, 2, 2, 64)	Kernel size = (1, 2, 2)
MaxPooling 3D	(1, 2, 2, 64)	-
Batch Normalization	(1, 2, 2, 64)	-
Convolutional 3D	(1, 2, 2, 64)	Kernel size = (1, 2, 2)
MaxPooling 3D	(1, 1, 1, 64)	-
Flatten	(64)	-
Batch Normalization	(64)	-
Dense	(1598)	Activation function = Tanh
Batch Normalization	(1598)	-
Dense	(1598)	Activation function = Tanh

Table S2. 3D-LSTM structure and characteristics. Notice that this network forecasts the wind speed, 2m dew point temperature and precipitation. The convolutional layers have a dropout of 0.4, and the recurrent layer has a drop out of 0.2 to avoid overfitting.

Type of Layer	Output	Characteristics
Convolutional 3D	(1, 7, 6, 32)	Kernel size = (56,2,2)
MaxPooling 3D	(1, 3, 3, 32)	-
Batch Normalization	(1, 3, 3, 32)	-
Convolutional 3D	(1, 2, 2, 64)	Kernel size = (1, 2, 2)
MaxPooling 3D	(1, 2, 2, 64)	-
Batch Normalization	(1, 2, 2, 64)	-

Convolutional LSTM 2D	(1, 2, 2, 64)	Kernel size = (1, 2, 2)
Flatten	(64)	-
Batch Normalization	(64)	-
Dense	(1598)	Activation function = Tanh
Batch Normalization	(1598)	-
Dense	(1598)	Activation function = Tanh

Section S2. WRF-Model parameterization setup

The model uses the Thompson microphysics scheme [1], which was improved by [2]. The model also uses the Grell-Freitas cumulus scheme ([3] modified by [4]) and the RRTMG long–short wave radiation ([5] modified by [6]). The subgrid-scale turbulent fluxes were resolved with the Smagorinsky 2D scheme [7], the YSU-PBL scheme was used for the boundary layer [8] and the surface fluxes were calculated using the Monin–Obukhov similarity theory [9].

Section S3. WRF-1D description

Before describing the WRF-1D procedure, it is important to clarify the difference between the executions for *Cundinamarca*. *Cundinamarca* was validated for 160 days and was then BIAS corrected with a trained neural network with 5 years of simulations run. This BIAS correction technique is described in this section.

The variables used are: relative humidity, 2m air temperature, 10m wind components (u and v), convective rain, nonconvective rain, downward shortwave radiation, surface longwave radiation, latent heat flux, sensible heat flux, vertical velocity and planetary boundary layer height. These variables were also subject to a Variance Inflation Factor (VIF) analysis before including them [10], and all were selected to be used in the neural net since the collinearity was less than 5 [11]. The variables were extracted from the WRF output as a time series from the pixel that was in the same location as each station. These variables were input into the network as a matrix in t = 1 (24h in the future), which refers to the future or the predicted day. This was combined with the relative humidity, 2m temperature, precipitation and 10m wind speed from each of the 8 stations evaluated in Cundinamarca. The station variables had null values, so an imputation technique was performed following Mogollon-Sotelo et al. [12]. These variables were included in the X-matrix at t = 0 (24h prior), which refers to the present day, not the future. In other words, the X-matrix was constructed with 14 variables, 10 from the WRF output as a time series in t=1 and 4 from the stations in t=0.

The Y-vector was constructed by a time series in every one of the five station variables in t=1 so that the output coincided with the WRF output. Thus, the network was trained multiple times, one for each station and one for each output variable, except for the precipitation. The precipitation had to be included by averaging the stations that were in a pixel of the model so that they could be evaluated [13]. Hence, this variable did not change per station but per pixel, different from the others that changed depending on the station, not the pixel. After the training was finished, 160 days were validated using the IOA, the Rho, the RMSE and three categorical parameters, the HIT, FAR and POC (see Celis et al.'s [14] Supplementary Material to see the equations and definitions), but only in the station sites. These categorical parameters were used for the FWI, calculated using the model output. It is important to mention that the three neural networks were designed using Tensorflow-2.4.1[15] and Keras-2.4.3 [16] libraries from Python-3.8.

Section S4. Forecast variables and data input/output

Table S3. Ranges of spectral indices used to categorize vegetation density (NDVI) and humidity (NDWI). Note that the moisture levels are expressed in terms of water stress and waterlogging.

Spectral index	Class ID
**NDVI**	Dense vegetation: 1 (1 <= NDVI > 0.6)<br>Moderate vegetation: 2 (0.6 <= NDVI > 0.4)<br>Low vegetation: 3 (0.4 <= NDVI > 0.2)<br>Nonvegetation (barren land/built up/ rock): 4 (0.2 <= NDVI > 0.0)<br>Nonvegetation (water): 5 (0.0 <= NDVI)
**NDWI**	Total canopy cover: 1 (1 <= NDWI > 0.8)<br>Very high canopy cover: 2 (0.8 <= NDWI > 0.6)<br>High canopy cover: 3 (0.6 <= NDWI > 0.4)<br>Mid–high canopy cover: 4 (0.4 <= NDWI > 0.2)<br>Average canopy cover: 5 (0.2 <= NDWI > 0.0)<br>Mid–low canopy cover: 6 (0.0 <= NDWI > -0.2)<br>Low canopy cover: 7 (-0.2 <= NDWI > -0.4)<br>Very low canopy cover: 8 (-0.4 <= NDWI > -0.6)<br>Almost absent canopy cover: 9 (-0.6 <= NDWI > -0.8)<br>Bare soil: 10 (-0.8 <= NDWI)

Table S4. Land cover classes and its correspondent ID category

**Land Cover Class**	**Class ID**
Water (DWAT/SWAT)	WAT = (DWAT: 1; SWAT: 2)
Clouds	CL: 3
Snow	SI: 4
Dense Forest/Dense Shrub	TCD: 5
Open Forest/Shrub	TCL: 6
Dense Shrub	SHR: 7
Dense Grassland/Open Shrum	GRS: 8
Sparse Grassland/Sparse Shrub	SPV: 9
Light soil/rocks/sand	OLL: 10
Dark soil/rocks/sand	OLD: 11
Shadowed/Low Illuminated	SV: 12

Vegetation	
Shadowed Soil/Burnt Areas	SS: 13
Nondata	NA: 0

Section S5. Global Vulnerability

Table S5. Multicriteria evaluation matrix of global vulnerability criteria of scientific references.

	**Social Vulnerability**									**Ecological Vulnerability**							
Reference	Occupation (WUI areas)	Adjacent	Proximity	Response capacity	Social and road infrastructure	Institutionality against risk	Land Use	Economic conditions	Demographic characteristics of population	Types of covers (adaptation to fire)	Strategic ecosystems	WUI	Soil characteristics	Fragmentation	Vegetal cover susceptibility	Endemism and threatened species	Recovery capacity
[17]	0	0	0	0	0	0	0	0	0	1	0	0	1	0	0	0	0
[18]	1	1	0	0	0	0	0	0	0	1	0	0	0	1	0	0	0
[19]	0	1	1	0	0	0	0	1	0	1	0	1	0	1	0	1	1
[20]	1	0	0	0	0	0	0	0	1	0	0	1	0	0	0	0	0
[21]	1	1	1	1	0	0	0	0	0	0	0	1	0	0	0	0	0
[22]	1	0	0	1	1	0	0	1	1	1	0	1	0	0	0	0	0
[23]	1	0	0	0	1	0	1	1	0	0	1	0	1	1	0	0	0
[24]	1	1	1	1	0	1	0	0	0	0	1	0	0	0	0	0	0
[25]	1	1	1	1	0	0	0	0	0	0	0	0	0	0	0	0	0
[26]	1	1	1	1	1	1	1	0	0	1	1	0	0	1	1	1	0
[27]	1	0	0	0	0	0	1	0	0	1	0	0	1	0		0	1
[28]	1	0	0	0	0	0	1	0	0	1	0	1	0	0	0	0	0
[29]	0	0	0	0	0	0	0	0	0	1	1	0	0	0	1	0	0
TOTAL	10	6	5	5	3	2	4	3	2	8	4	5	3	4	2	2	2

Section S6. Susceptibility categorization and gradient

Soil cover categorization method : Thereafter, we determined the zones inside the study area where those practices would be more effective, and where they are able to be established and standardized. Thus, we used the official land cover distribution for *Colombia*, constructed following the Corine land cover (CLC) methodology of Cruz et al [30]. We used the land cover for the periods of 2000-2002, 2010-2012 and 2018 (hereafter 2000, 2010 and 2018, respectively). It is important to note that we only used the level 3 CLC information and subdivided the study areas into 44 (*Cundinamarca*) and 47 (*Magdalena*) types of covers. An example of this method for the 2018 land cover is explained below. Fig. SM1 shows 13 macrozones excluding areas with urban settlements and with the presence of water. The division level was chosen to recommend practices that can be used according to the identified vegetation material. With the segregated information, an 8-year period (2010-2018) analysis was performed in order to produce medium- and long-term strategies to include in the protocol. This analysis was based on the sustainability of land cover in regard to wildfires. Hence, we calculated the susceptibility index of the cover to burn proposed by Paramo-Rocha [31] for every cover type in the CLC level 3 subdivision. This index uses the type of fuel, its duration and its total load as categories to establish susceptibility for every year.

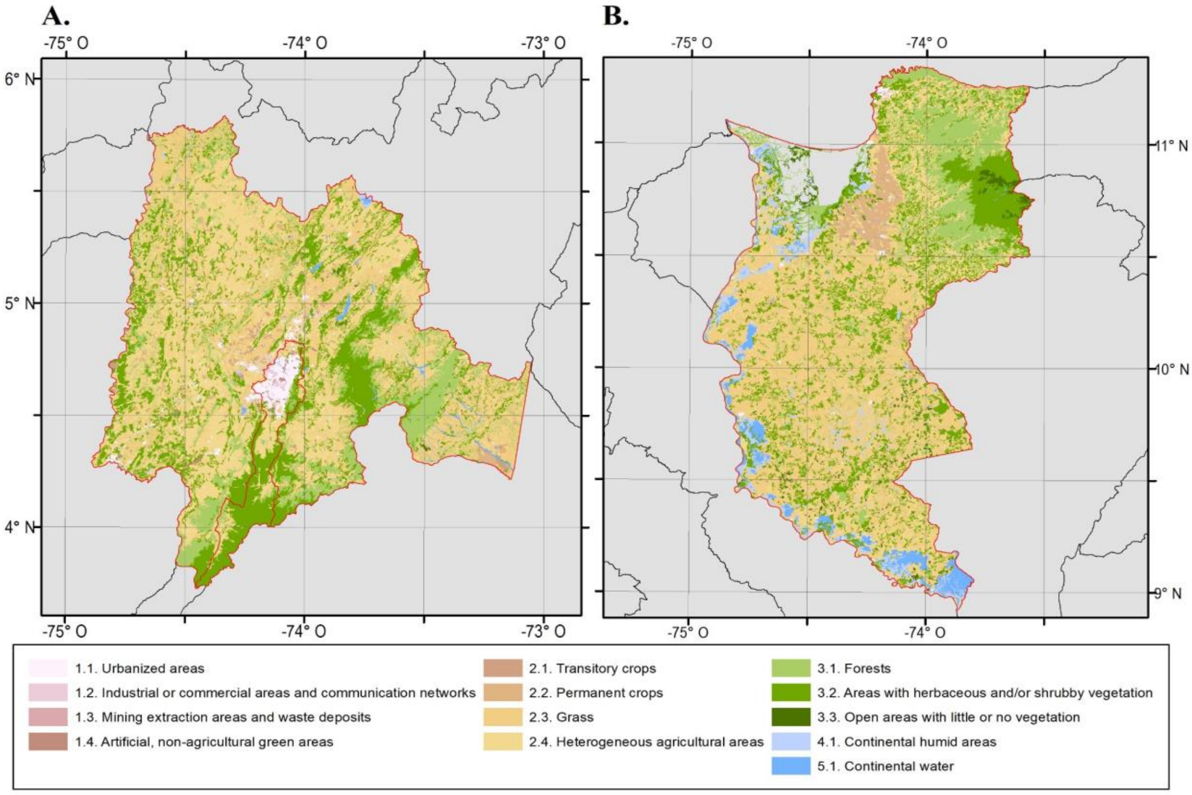

Figure S1. Layout of surface cover distribution for the two study areas into 13 subdivisions in 2018, for A) *Cundinamarca* and B) *Magdalena*. The strong red line represents the departmental border of each study area, the reddish shades show urban areas and constructions, the brown shades indicate large crop areas, the orange shades represent pastures and crops, the green shades show natural forests and blue shades represent presence of water.

Soil cover categorization results: Analyzing changes in the level of susceptibility to fire due to the coverage change, four ranges of positive and negative change were defined to address all possible changes from levels zero, low, moderate, high and extreme susceptibility (Fig. SM2). For instance, a

level 1 positive change shows a place where the ground increased in one level of susceptibility, i.e., from no susceptibility to low, from low to moderate, from moderate to high or from high to extreme. Accordingly, a level 4 positive change indicates that the land went from zero susceptibility to extreme susceptibility in the time window observation. Negative changes indicate a decrease in susceptibility, and its ranges follow the same described structure. Table SM6 shows all changes in the area and percentage of the susceptibility to fire due to changes in the coverage from 2010 to 2018. Positive and low-level negative changes are the most common. However, negative changes in susceptibility are strongly related to the expansion of cities, which decreases the susceptibility of a coverage to ignite, but it is not necessarily an optimal result in regard to soil reparation, because the land cover is being lost. In terms of the positive changes, the practices 1.1, 2.1, 2.2 and 2.3 can be used, depending on the LCT, the susceptibility change factor and the susceptibility identified.

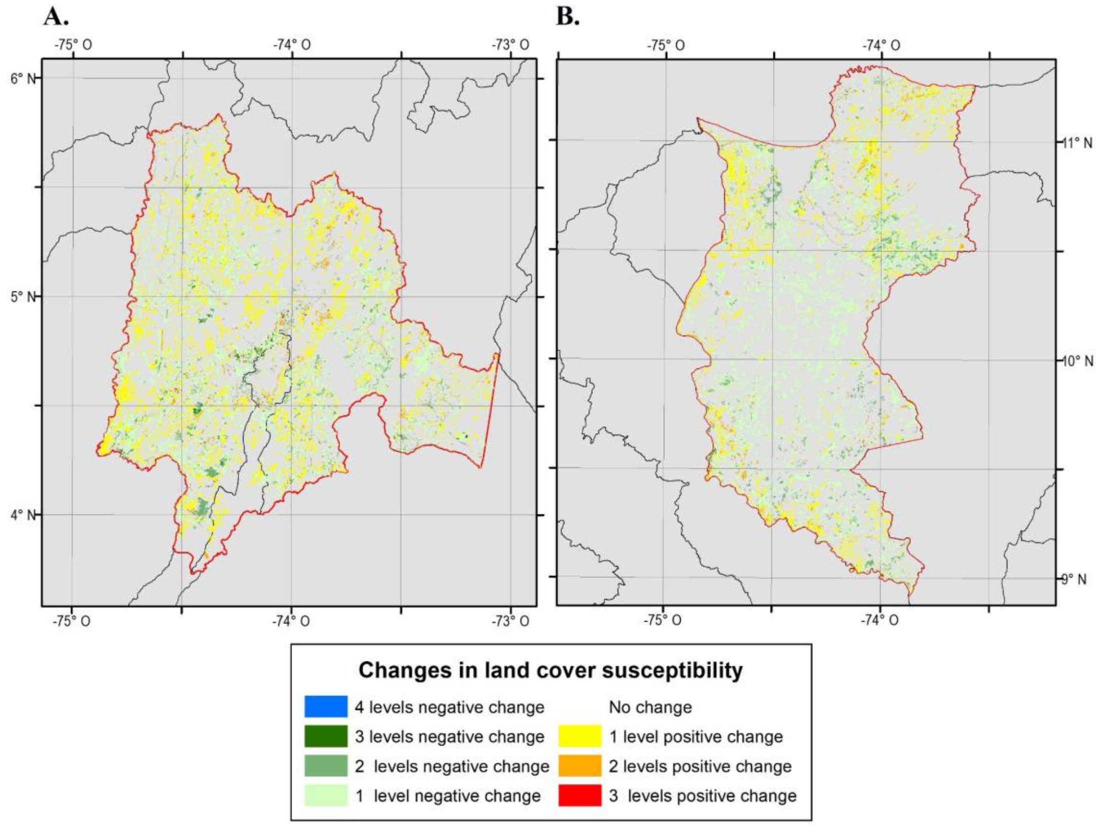

Figure S2. Change in the susceptibility of covers to fire of the two study areas between 2010 and 2018 for A) *Cundinamarca* and B) *Magdalena*. Positive level changes, in warm colors, represent an increase in susceptibility and negative changes, and in cold colors represent a decrease in the susceptibility of the cover. No change in coverage susceptibility is represented by locations with no color.

The *Cundinamarca* and *Magdalena* areas presented a decrease in land cover susceptibility to fire, which implies more resistance to wildfires (Fig. SM2, Table SM6). Nonetheless, it is always necessary to determine the sensibility before adapting one management strategy, and in cases where the zone has a high ecological importance, it is better to make in situ measurements, which would allow a better understanding of the environmental conditions and their related changes. Most of the positive changes, on the other hand, are related to level 1 of change, especially in *Cundinamarca*, meaning that *Cundinamarca* has a stronger susceptibility to develop wildfires. This could suggest that *Cundinamarca* has transformed many forest and pasture areas to crop and livestock areas. Following the same idea, it is recommended that the application of the SMP starts on the red dots of Fig. SM2, which corresponds

to 1.678 Ha and 625 Ha in *Cundinamarca* and *Magdalena*, respectively. This is suggested because the susceptibility is very high, and the politics do not seem to focus on preventing the changes that are being produced in this area.

These red zones need to be intervened as soon as possible. First, it is important to apply the SMP 2.2-2.1-2.3 consecutively in zones where wildfires have previously appeared, action 2.2 serves to reduce the risk of a new anthropogenic ignition, while strategies 2.1 and 2.3 focus on recovering the vegetation cover to mitigate the erosion and mass movements. On the other hand, it is also important to make vegetation replacements as proposed in action 1.1, since this would reduce the risk by taking away the vegetation with a higher probability of producing and maintaining a fire (this works if there has been no recent wildfires). Nevertheless, this vegetation change has to take into account the geographic location and the ecological characteristics of the area. In the areas where a positive range of one for both cities is determined, the SMP 1.2 and 1.3 are adequate, since they focus on community edaphic governance. This can develop a greater control of the soil and avoid a greater change in vegetation cover, which could be highly impactful due to the increase in fire risk and ignition. In summary, high susceptibility areas can be managed in the long term if the measures of Table 4 are followed, since improving the vegetation cover (which would also adapt, since the plan guarantees that those covers are related to the ones previously established), reducing the erosion and conserving the basic properties of the soil could avoid an increase in the risk.

Table S6. Percentage and area description of the change in the susceptibility of the cover to fire in 2010-2018 for the two study areas. The area indicates the hectares that went through each level of change. The change represents the percentage of change in that level with respect to the total territory.

	***Cundinamarca* 2010-2018**		***Magdalena* 2010-2018**	
	**Area (Ha)**	**Change (%)**	**Area**	**Change (%)**

**No Change**	1729374.48	72.2%	1765401.98	76.7%
**1 level (+)**	268035.61	11.2%	139967.44	6.1%
**2 level (+)**	23974.99	1.0%	21272.36	0.9%
**3 level (+)**	1677.83	0.1%	624.80	0.0%
**4 level (+)**	0.00	0.0%	0.00	0.0%
**1 level (-)**	311471.52	13.0%	319031.10	13.9%
**2 level (-)**	55027.21	2.3%	53876.63	2.3%
**3 level (-)**	6433.37	0.3%	1322.18	0.1%
**4 level (-)**	0.00367	0.0%	0.00	0.0%

Section 7. Soil Management practices analysis

Table S7. Shows the pre-fire practices selected from the review of governmental management documents and guides available in different Latin American countries

**Country**	**Reference**	**Prefire practices**							
		Wildfire protection trails	Controlled use of fire in agriculture	Educational actions	Removal of forest fuels	Policy actions	Soil moisture maintenance	Use of organic waste as fertilizer	Preventive forestry
México	[32]	0	0	0	0	0	0	0	0
	[33]	0	1	1	0	1	0	0	1
	[34]	0	0	1	1	0	0	0	0
	[35]	0	1	1	1	1	0	0	1
Colombia	[36]	0	1	0	1	0	1	0	0
	[37]	1	0	0	1	0	0	0	1
	[38]	0	1	1	0	0	0	1	1
	[39]	0	1	1	0	0	0	1	0
Perú	[40]	0	0	1	0	0	0	0	0
	[41]	0	0	1	1	0	0	0	0

	[42]	0	0	0	1	0	0	0	0
Chile	[43]	0	0	1	1	0	0	0	0
	[44]	0	0	0	0	0	0	0	0
	[45]	0	0	1	0	0	0	0	0
	[46]	0	0	0	1	0	0	0	0
Total		1	6	9	10	2	1	2	5

Table S8. Shows the postfire practices selected from the review of governmental management documents and guides available in the different Latin American countries

**Country**	**Reference**	**Postfire practices**							
		Vegetation recovery of areas damaged by forest fires	Soil protection in high slopes	Forest stand cleaning	Trail and road closure	Planting of herbaceous and shrubby plants	Ecological restoration	Soil control in wildfires	Soil or fuel indicators
México	[32]	1	1	1	1	0	0	0	0
	[33]	0	0	0	0	1	1	0	0
	[34]	0	0	0	0	0	0	0	0
	[35]	0	0	0	0	0	0	0	0
Colombia	[36]	1	1	0	0	1	0	0	0
	[37]	0	0	0	0	0	0	0	0
	[38]	1	0	0	0	0	0	0	0
	[39]	0	0	0	0	0	0	0	0
Perú	[40]	0	0	0	0	0	0	0	0
	[41]	1	0	0	0	0	0	0	0
	[42]	0	0	0	0	0	0	1	0
Chile	[43]	1	0	1	0	0	0	1	1
	[44]	0	0	0	0	0	0	1	0

	[45]	0	0	0	0	0	0	0	0
	[46]	0	0	0	0	0	0	0	0
Total		5	2	3	1	2	1	3	1

Section S8. Meteorological validation

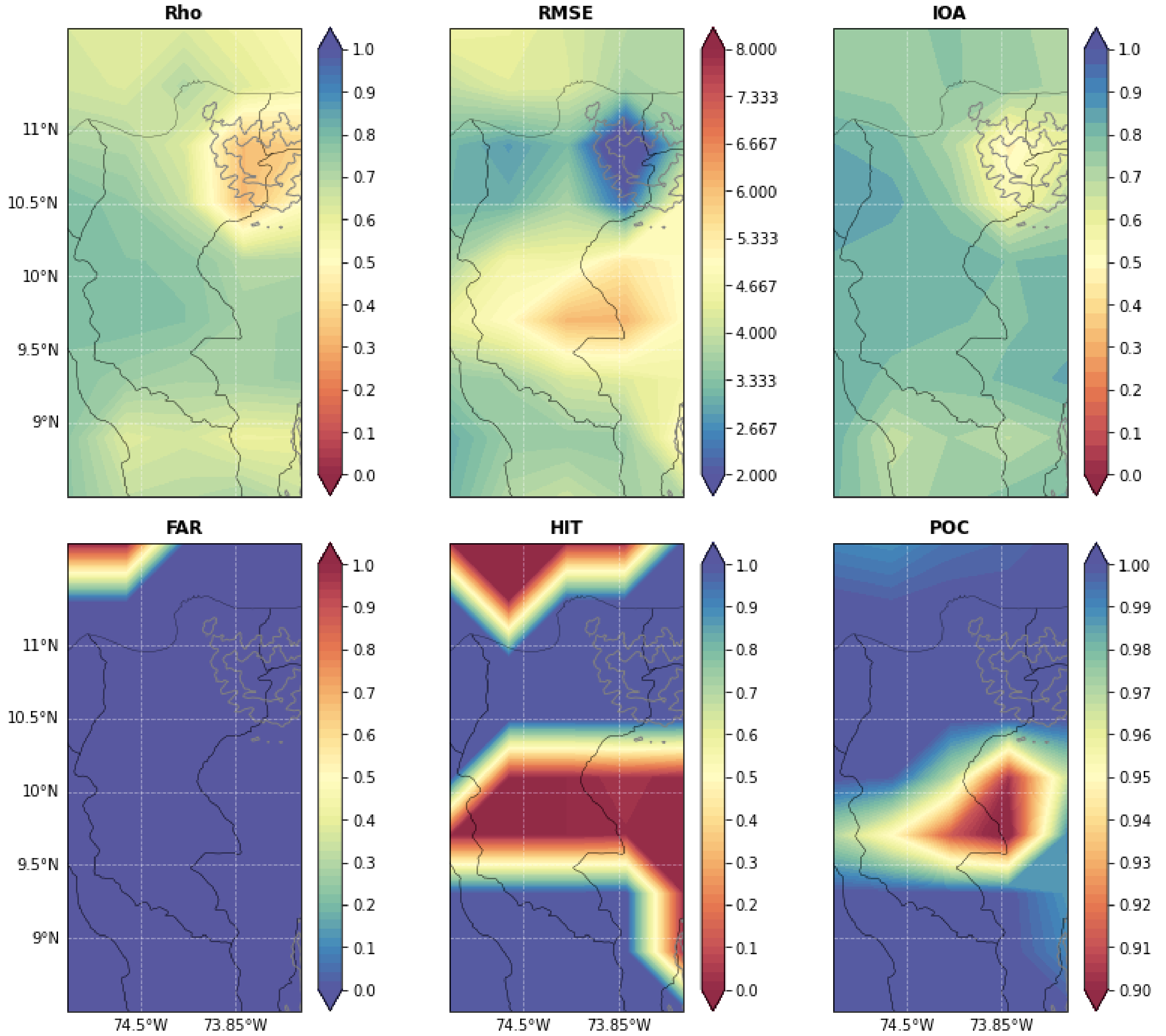

Figure S3. Statistical parameters of the neural net for *Magdalena*. The title of each plot represents the calculated parameter, and the panels have different colorbars. The gray contours indicate the *Andean* Mountain ranges.

References

1. Thompson, G.; Field, P.R.; Rasmussen, R.M.; Hall, W.D. Explicit forecasts of winter precipitation using an improved bulk microphysics scheme. Part II: Implementation of a new snow parameterization. *Mon. Weather Rev.* **2008**, *136*, 5095-5115. https://doi.org/10.1175/2008MWR2387.1
2. Iversen, E.C.; Thompson, G;, Nygaard, B.E. Improvements to melting snow behavior in a bulk microphysics scheme. *Atmos. Res.* **2021**, *253*, 105471, https://doi.org/10.1016/j.atmosres.2021.105471
3. Grell, G.A.; Devenyi, D. A scale and aerosol aware stochastic convective parameterization for

weather and air quality modeling. *Atmos. Chem. Phys*. **2014**, *14,* 5233-5250. https://doi.org/10.5194/acp-14-5233-2014
4. Grell, G.A.; Freitas, S. R.A generalized approach to parameterizing convection combining ensemble and data assimilation techniques. *Geophys. Res. Lett.* **2002**, *29,* 1693. https://doi.org/10.1029/2002GL015311
5. Mlawer, E. J.; Taubman, S. J.; Brown, P. D.; Iacono, M. J.; Clough, S. A. Radiative transfer for inhomogeneous atmospheres: RRTM, a validated correlated-k model for the longwave. *Journal of Geophysical Research: Atmospheres.* **1997**, *102,* 16663–16682, https://doi.org/10.1029/97JD00237
6. Iacono, M. J.; Delamere, J. S.; Mlawer, E. J.; Shephard, M. W.; Clough, S. A.; Collins, W. D. Radiative forcing by long-lived greenhouse gases: Calculations with the AER radiative transfer models. *Journal of Geophysical Research: Atmospheres.* **2008**, *113,* D13, https://doi.org/10.1029/2008JD009944
7. Ghahremanloo, M.; Lops, Y.; Choi, Y.; Jung, J.; Mousevinezhad, S.; Hammond, D. A comprehensive study of the COVID-19 impact on $PM_{2.5}$ levels over the contiguous United States: A deep learning approach. *Atmos Environ* **2022,** 118944. https://doi.org/10.1016/j.atmosenv.2022.118944
8. Smagorinsky, J. General Circulation Experiments With the primitive equations: I. The Basic Experiment. Monthly Weather Review **1963**, *91*, 99-164, https://doi.org/10.1175/1520-0493(1963)091<0099:GCEWTP>2.3.CO;2
9. Hong, S.; Noh, Y.; Dudhia, J. A New Vertical Diffusion Package with an Explicit Treatment of Entrainment Processes. Monthly Weather Review. **2006**, *134,* 2318-2341, 10.1175/MWR3199.1
10. Monin, A.; Obukhov, A. Basic Laws of Turbulent Mixing in the Surface Layer of the Atmosphere.Tr. *Akad. Nauk SSSR Geophiz. Inst.* **1954**, *24*(151), 163-187.
11. Kline, R.B. Principles and Practice of Structural Equation Modeling. 4rd ed.; Publisher: Guilford Publications, New York, London, USA, **2015**; pp. 534. https://www.researchgate.net/publication/361910413_Principles_and_Practice_of_Structural_Equation_Modeling
12. Mogollón-Sotelo, C.; Casallas, A.; Vidal, S.; Celis, N.; Ferro, C.; Belalcázar, L.C. A support vector machine model to forecast ground-level $PM_{2.5}$ in a highly populated city with complex terrain. *Air Qual. Atmos. Health* **2021**, *14*, 399-409, https://doi.org/10.1007/s11869-020-00945-0
13. Jeworrek, J.; West, G.; Stull, R. WRF Precipitation Performance and Predictability for Systematically Varied Parameterizations over Complex Terrain. *Weather and Forecasting.* **2021,** *36*(3), 893-913, https://doi.org/10.1175/WAF-D-20-0195.1
14. Celis, N.; Casallas, A.; Lopez-Barrera, E.A.; Martínez, H.; Peña-Rincón, C.A.; Arenas, R.; Ferro, C. Design of an Early Alert System for PM2.5 through a stochastic method and machine learning models. *Environ. Sci. Pol.* **2022**, *127*, 241-252, https://doi.org/10.1016/j.envsci.2021.10.030
15. Abadi, M.; Agarwal, A.; Barham, P.; Brevdo, E.; Chen, Z.; Citro, C.; Corrado, G. S.; Davis, A.; Dean, J.; Devin, M. ; et al.. TensorFlow: Large-scale machine learning on heterogeneous systems, **2015**. vo.2.4.1. (Version 0.2.4.1). *Tensorflow*. https://doi.org/10.48550/arXiv.1603.04467
16. GitHub.keras-team/keras. vo.2.4.3. Keras. Available online:https://github.com/keras-team/keras. (accessed on 11 November 2021)
17. Duguy, B.; Alloza, J. A.; Baeza, M. J.; De la Riva, J.; Echeverría, M., Ibarra, P.; Llovet, J; Perez, F.; Rovira, P; Vallejo, R. V. Modelling the ecological vulnerability to forest fires in Mediterranean ecosystems using geographic information technologies.*Environmental management.* **2012**, 50(6), 1012-1026.https://doi.org/10.1007/s00267-012-9933-3
18. Vargas-Sanabria, D.; Campos-Vargas, C. Modelo de vulnerabilidad ante incendios forestales para el Área de Conservación Guanacaste, Costa Rica. *Cuadernos de Investigación UNED,* **2018,** 10(2), 435-446. http://dx.doi.org/10.22458/urj.v10i2.2173
19. Chuvieco, E.; Martínez, S.; Román, M. V.; Hantson, S.; Pettinari, M. L. Integration of ecological and socio-economic factors to assess global vulnerability to wildfire. *Global Ecology and Biogeography*, **2014**, 23(2), 245-258. https://doi.org/10.1111/geb.12095
20. Wigtil, G.; Hammer, R. B.; Kline, J. D.; Mockrin, M. H.; Stewart, S. I.; Roper, D.; Radeloff, V. C. (2016). Places where wildfire potential and social vulnerability coincide in the coterminous United States. *International Journal of Wildland Fire,* **2016**, 25(8), 896-908.

21. Ramos, W. H. (2014). Factores de vulnerabilidad ante los incendios forestales en las provincias de Alicante y Valencia. *Investigaciones Geográficas (España)* **2014**, 62, 143-161. https://doi.org/10.14198/INGEO2014.61.10
22. Cutter, S. L.; Mitchell, J. T.; Scott, M. S. Revealing the vulnerability of people and places: A case study of Georgetown County, South Carolina. In: Hazards Vulnerability and Environmental Justice. 1St Edition. **2000.** Publisher: *Routledge*, *Annals of the association of American Geographers*, London, United Kingdom, 90(4), 713-737.
23. Chuvieco, E.; Aguado, I.; Jurdao, S.; Pettinari, M. L.; Yebra, M., Salas; Hantson S.; De la Riva A.J; Ibarra, B. P; Rodrigues, B.M. Integrating geospatial information into fire risk assessment. *International journal of wildland fire*, **2012**, 23(5), 606-619. https://doi.org/10.1071/WF12052
24. Tedim, F. O contributo da vulnerabilidade na redução do risco de incêndio florestal. In: Riscos Naturais, Antrópicos e Mistos. **2013**. Publisher: Lourenço, L.; Mateus, M.A. Eds. Departamento de Geografia da Faculdade de Letras da Universidade de Coimbra, Coimbra, Portugal. pp. 653-666.
25. Tedim, F.; Leone, V.; Amraoui, M.; Bouillon, C.; Coughlan, M.R.; Delogu, G.M.; Fernandes, P.M.; Ferreira, C.; McCaffrey, S; McGee, T.K. et al. Defining extreme wildfire events: difficulties, challenges, and impacts. *Fire*, **2018.** 1(1), 9. https://doi.org/10.3390/fire10
26. IDEAM. Protocolo para la realización de mapas de zonificación de riesgos a incendios de la cobertura vegetal - Escala 1:100.000. **2011.** Publisher: Instituto de Hidrología, Meteorología y Estudios Ambientales-IDEAM. Bogotá, Colombia. pp. 109.
27. Román, J.G.; Álvarez-Garay, B. Dinámica de incendios en el Área de Conservación Guanacaste 1997-2017: perspectivas ecológicas para el manejo integral del fuego. *Perspectivas Rurales Nueva Época*, **2018,** 16(31), 51-70. https://doi.org/10.15359/prne.16-31.4.
28. Miranda, A., Carrasco, J., González, M., Pais, C., Lara, A., Altamirano, Weintraub, A.; Syphard, A. D. Evidence-based mapping of the wildland-urban interface to better identify human communities threatened by wildfires. *Environmental Research Letters*, **2020.** 15(9), 094069. https://iopscience.iop.org/article/10.1088/1748-9326/ab9be5/meta
29. Moreno, A.; Montealegre, F; Vargas Y. Propuesta metodológica para la evaluación de la susceptibilidad de la cobertura vegetal a la ocurrencia de incendios forestales usando imágenes SENTINEL-2B. **2021.** Master Thesis. Master in information management and geospatial technologies. Universidad Sergio Arboleda. Bogotá, Colombia. pp. 27
30. Cruz, A.; Acosta, H.; Barajas, P.; Castellanos, H.; Ciontescu, N.;Corredor, L.; Espejo, C.; Huertas, C.; Martin, C.; Ramírez, C.; Ramírez, K.; Rodríguez, A.; Rodríguez, O. Catálogo de patrones de coberturas de la tierra Colombia. Technical Report IDEAM. **2012.** Publisher: Instituto de Hidrología, Meteorología y Estudios Ambientales. Bogotá, Colombia. pp. 8
31. Páramo-Rocha, G.E. Susceptibilidad de las coberturas vegetales de Colombia al fuego. In Incendios de la cobertura vegetal en Colombia, Parra Lara, A. D. C.; Bernal Toro, F. H.; Armenteras Pascual, D.; González Alonso, F.; Morales Rivas, M.; Pabón Caicedo, D. J.; Páramo Rocha, G. E. Publisher: Universidad Autónoma de Occidente. **2011**. Cali, Colombia. pp. 73- 143.
32. Comisión Nacional Forestal de México. Incendios forestales. *Guía práctica para comunicadores*. Technical document of The Nature Conservancy y la Comisión Nacional Forestal. **2010.** Publisher: Efecto Marketing. Guadalajara, Jalisco México. pp.56. http://www.conafor.gob.mx:8080/documentos/docs/10/236Gu%C3%ADa%20pr%C3%A1ctica%20para%20comunicadores%20-%20Incendios%20Forestales.pdf
33. Flores, J.; Flores, A.; Lomeli, M.; García, J. Plan Estatal de Manejo del Fuego en el Estado de Jalisco. Primera etapa de estudio. Publisher: *Secretaría de medio ambiente y desarrollo territorial.* **2018.** Guadalajara, Jalisco, México, pp. 145. https://transparencia.info.jalisco.gob.mx/sites/default/files/2018.%20PrimeraEtapaPlanManejoFuegoT1.pdf .
34. Comisión Nacional Forestal- CONAFOR-. Programa de manejo del fuego 2019 gerencia de manejo del fuego contenido. Publisher: Secretaría de Medio Ambiente y Recursos Naturales. Technical document. **2019**. México D.F. México. pp.63 https://www.gob.mx/cms/uploads/attachment/file/464834/PROGRAMA_DE_MANEJO_DEL_FUEGO_2019.pdf
35. Comisión Nacional Forestal- CONAFOR-. Programa de manejo del fuego 2020-2024. Coordinación General de Conservación y Restauración. Publisher:: *Gerencia de manejo del fuego*.

Technical document. **2020.** México D.F. México. pp.101. https://idefor.cnf.gob.mx/documents/829/download.
36. Hernández, H. M. Lo que usted debe saber sobre incendios de cobertura vegetal. Publisher: *Unidad nacional para la gestión de riesgo de desastres- UNGRD.* Technical document.. **2019.** Bogotá. Colombia. pp.44. .https://repositorio.gestiondelriesgo.gov.co/bitstream/handle/20.500.11762/28309/Cartilla_Incendios_2019-.pdf?sequence=4#:~:text=Si%20se%20origina%20un%20incendio,p%C3%A9rdidas%20ecol%C3%B3gicas%2C%20econ%C3%B3micas%20y%20sociales.
37. Torres, C. E. Documento técnico de soporte a amenazas por incendios forestales. *Plan de ordenamiento territorial de Bogotá.* Publisher: Instituto Distrital de Gestión de Riesgos y Cambio Climático IDIGER. **2019.** Bogotá. Colombia. pp.73. https://www.sdp.gov.co/sites/default/files/POT/4-DOCUMENTO_TECNICO_DE_SOPORTE_14-06-19/DT04_Anexo14_MapadeAmenaza_porIncendiosForestales.pdf
38. IDEAM. Informe anual sobre el estado del medio ambiente y los recursos naturales renovables en Colombia: bosques - 2009. Publisher: Instituto de Hidrología, Meteorología y Estudios Ambientales- IDEAM. **2010.** Bogotá. Colombia. pp.235 http://documentacion.ideam.gov.co/cgi-bin/koha/opac-detail.pl?biblionumber=9114
39. CVS. Protocolo local de estadísticas, análisis y medidas de manejo de incendios forestales en ecosistemas estratégicos del departamento de Córdoba. Publisher: Corporación Autónoma Regional de los Valles del Sinú y del San Jorge. **2019.** Córdoba, Colombia. pp.235 protocolo-local-de-incendios-forestales.pdf
40. SERFOR. Plan de prevención y reducción de riesgos de incendios forestales. Publisher: Servicio Nacional Forestal y de Fauna Silvestre - Ministerio de Agricultura y riego de Perú. **2018**. Lima. Perú. pp.60. https://www.serfor.gob.pe/portal/wp-content/uploads/2018/12/Plan-de-prevenci%C3%B3n-y-reducci%C3%B3n-de-riesgos-de-incendios-forestales.pdf
41. SERNANP. Estrategia de Gestión del Riesgo de Incendio Forestal en el Sistema Nacional de Áreas Naturales Protegidas por el Estado. Publisher: Servicio Nacional de áreas naturales protegidas por el estado - Ministerio de Agricultura y riego de Perú. **2018**. Lima. Perú. pp.84. https://cdn.www.gob.pe/uploads/document/file/475395/estrategia_incendio-forestal-baja.pdf
42. Jara, J.L.; Florez, J.; Mujica, O.; Chalan, I.; Silva., J. Manual para el Control de Incendios Forestales- SERNANP-Parque Nacional del Manu. Publisher: Servicio Nacional de áreas naturales protegidas por el estado-SERNAP, Sociedad Zoológica de Francfort. **2016**. Cusco. Perú. pp.172. https://peru.fzs.org/noticias/manual-para-el-control-de-incendios-forestales-sernanp-parque-nacional-del-manu/
43. Fernández, I.; Morales, N.; Olivares, L.; Salvatierra, J.; Gómez, M.; Montenegro, G. Restauración ecológica para ecosistemas nativos afectados por incendios forestales. 1st edition. Publisher: Pontificia Universidad Católica de Chile. **2010**. Santiago. Chile. pp.149. https://www.conaf.cl/wp-content/files_mf/1363716217res_baja.pdf
44. CONAF- Corporación Nacional Forestal. La Motosierra. Working Paper 565. Publisher: Gerencia de Manejo del Fuego Departamento de Desarrollo y Normalización - *Ministerio de Agricultura.* **2011**. Santiago. Chile. pp. 61. https://www.conaf.cl/wp-content/files_mf/1363718291LAMOTOSIERRAmanual.pdf
45. Cornejo, B.; Cuq, V.; González, Á.; Ortega, M.; Saballa, P. Manual con Medidas para la Prevención de Incendios Forestales Región Coquimbo. Working Paper 566. Publisher: *Corporación nacional forestal -CONAF-* Gerencia de Manejo del Fuego Departamento de Desarrollo y Normalización - *Ministerio de Agricultura.* **2011**. Santiago. Chile. pp. 68. https://bibliotecadigital.ciren.cl/bitstream/handle/20.500.13082/29335/manual%20medidas%20prevenci%c3%b3n%20IV.pdf?sequence=1&isAllowed=y
46. Ministerio de Agricultura. Decreto 276: Reglamento sobre Roce a Fuego. **2013**. pp. 4. http://extwprlegs1.fao.org/docs/pdf/chi207239.pdf
